# Lymphoepithelial – Like Carcinoma with Papillary Transitional Cell Carcinoma of the Urinary Bladder Associated with Carcinoma in situ Changes of the Urothelium; A Case Report and Review of Literature

**DOI:** 10.30699/IJP.14.2.156

**Published:** 2019-06-10

**Authors:** Yahya Attaran, Simin Moghdam, Ahmad Monabati, Reza Sarkeshikian

**Affiliations:** 1 *M.D, FCAP, Assistant professor of Pathology, Department of Pathology, Shiraz University of Medical Sciences, Shiraz, Iran *; 2 *M.D, Department of Pathology, Beheshti Hospital, Shiraz, Iran *; 3 *M.D, Professor of Pathology, Department of Pathology and Hematology Research Center, Shiraz University of Medical sciences, Shiraz, Iran *; 4 *M.D, Pasteur Pathobiology Laboratory, Shiraz, Iran*

**Keywords:** Urinary Bladder, like carcinoma, Carcinoma in situ

## Abstract

Lymphoepithelial - like carcinoma, is rarely recognized in the urinary bladder and less commonly occurs with papillary transitional cell carcinoma i.e. mixed pattern. Also, less uncommon is the occurrence of carcinoma in situ changes in the adjacent urothelium of these tumors. Here, a case of lymphoepithelial – like carcinoma and papillary transitional cell carcinoma associated with carcinoma in situ changes of urothelium of the urinary bladder has been reported the prognosis of this type of malignancy as well as its management will be discussed. Meanwhile, immunohistochemical stains have been carried out to differentiate it from lymphoma of the urinary bladder and the findings will be discussed.

## Introduction

Lymphoepithelioma is a form of undifferentiated carcinoma first described in the nasopharynx, and is more common in young Asian patients (1, 3). Histologically, this type of malignancy is characterized by the syncytial growth of malignant epithelial cells infiltrated by lymphoid cells (1). In this location, this type of malignancy has a close pathogenic link to Epstein – Barr Virus (1).

 Lymphoepithelial–like carcinoma with similar histology has been described in a variety of organs including the salivary glands, thymus, lung, skin, stomach, uterine cervix, breast, prostate, and urinary tract (1, 3). Some of these tumors have pathogenic link to EBV-Virus (1, 3). In the urinary tract, though rare, lymphoepithelial like carcinomas are reported in urinary bladder, but isolated cases of this tumor have been reported in the renal pelvis, ureter and urethra (3). Lymphoepithelial – like carcinoma accompanied by transitional cell carcinoma has been referred to mixed cases (3). Also, more uncommon is mixed cases of Lymphoepithelial – like carcinoma and Transitional cell carcinoma associated with carcinoma in situ of the adjacent urothelium. The most controversial issue is the prognosis and approach to the patient with Lymphoepithelial-Like carcinoma of urinary bladder. It has been suggested that patients with this type of malignancy have more favorable prognosis compared to the conventional invasive urothelial carcinoma as long as it is single (4, 6, 7, 9, 14-16). However, if this type of malignancy is associated with carcinoma in situ, the mode of treatment will be different. Whereas some people believe that more radical approach should be considered (3), the reason of such aggressive approach will be discussed. Meanwhile, Immunohistochemical stains have been suggested to differentiate this tumor from lymphoma of the urinary bladder (13).

## Case Reports

A fifty-eight (58) year-old male came to the hospital with gross and total hematuria since three months prior to admission. Cystoscopy was done, and it revealed a large three (3) cm fungating mass in the right side of the urinary bladder extending to the dome of the bladder. The mass was resected by transurethral resection (TURB) and send for pathology. Past history revealed that he was addicted to opium for many years and he was on Methadone. Routine H&E, PAS\ and imunohisto-chemical stains for cytokeratins CK7, CK20, P63, Alpha-Methyl –acyl – COA racemase, Thyroid Transcription Factor-1(TTF-1), Epstein –Barr Virus latent Membrane protein (LMP), and CD30 were carried out. Meanwhile, 103 cases of Transitional cell carcinoma (TURB) retrieved from the pathology archive and reviewed while looking for foci of Lymphoepithelial –like carcinoma (LELC), foci of carcinoma in situ changes in adjacent urothelium, muscular invasion and tumor grade. There was no ethical concern about the archives tissues; however, permission was taken to work on the current sample. 

**Pathological Findings**

**Figure 1:**
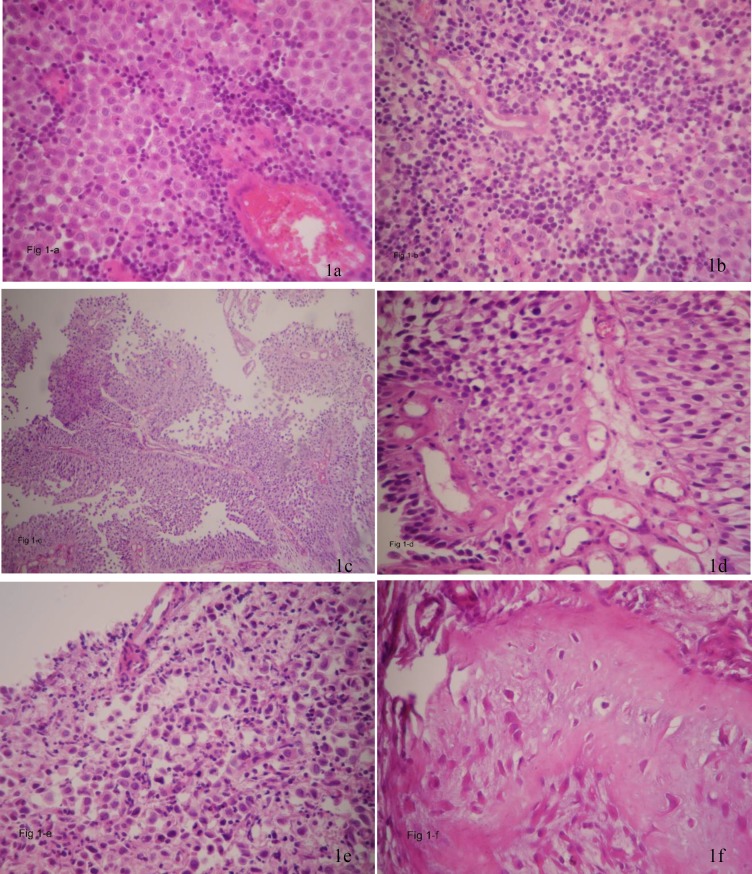
a & b) Show sheaths of syncitial epithelial cells between which are sprinkled lymphoid cells mainly lymphocytes (H&E, 25X ). c & d) Photomicrographs show papillary transitional cell carcinoma with several papillary structures lined by laryes of transitional cells (H&E, 4X). d) Higher power (H&E, 40x). e) Shows submucosal invasion by malignant cells having anaplastic nuclei and frequent mitosis (H&E, 40X). f) Shows muscular invasion by highly malignant cells (H&E, 40X)

**Figure 2:**
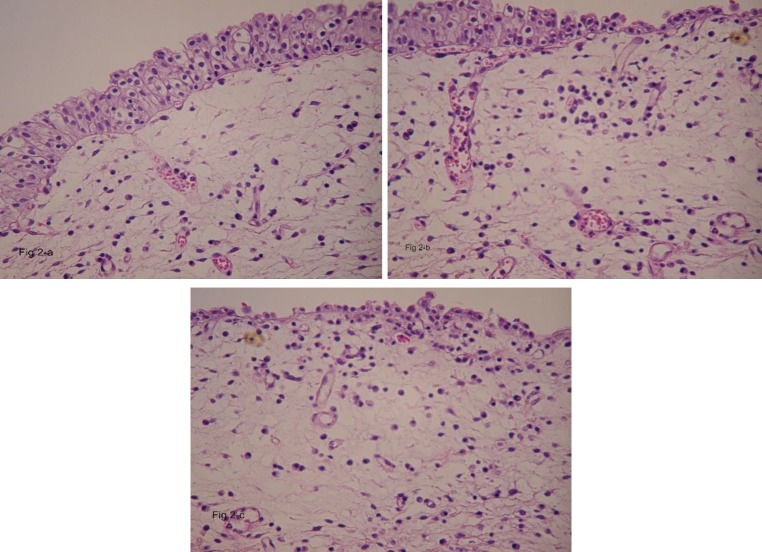
a, b, & c), Photomicrographs show carcinoma in situ changes of urothelium without papillary formation and multi-layering proliferation of malignant transitional cells, the individual cell shows nuclear anaplasia, loss of their polarity through the entire mucosal thickness, the submucosa is edematous, with dilated congested vessels and chronic inflammation (a & b H&E, 25X), c) Shows the same histological features and possible micro invasion by carcinoma in situ (arrows) (H&E, 25X)

**Figure 3:**
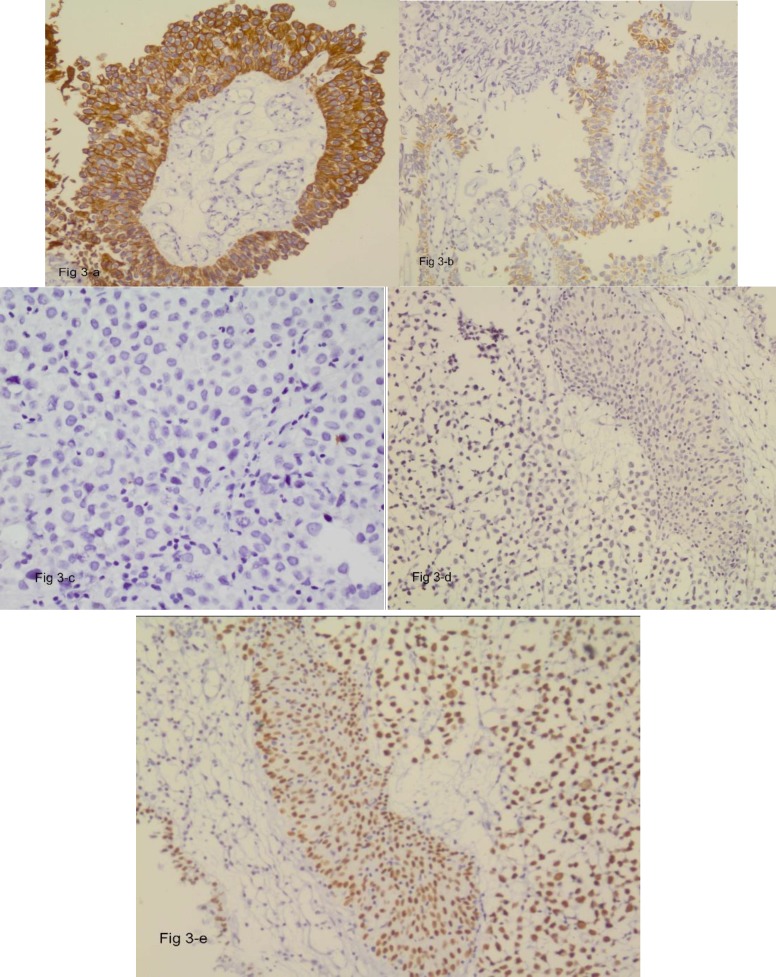
a ,b ,c ,d,& e ), immunohistochemical stains show a) Intense cytoplamic staining with CK 7 (25X), b) Focal positivity with CK 20 of transitional cells indicative of dysplastic changes (10X) , negative staining with LMP for EBV (c & d, 25X) and diffuse, intensely nuclear staining for P 63 in both Transitional and syncytial epithelial cells (e, 10X)

H&E sections from TUR-B specimen showed foci of syncytial epithelial cells with large hyperchromatic nuclei and a large amount of cytoplasm, sprinkled in between were chronic inflammatory cells mainly lymphocytes, Figure 1 (a & b). In adjacent areas, sections showed papillary transitional cell carcinoma in which the papillary structures were lined by multilayered transitional cells with large pleomorphic anaplastic nuclei (Figure 1c & d). There was submucosal invasion by highly malignant cells with large pleomorphic nuclei and frequent mitotic figures i.e. high grade (grade III), focally invaded muscular layer (Figure 1e & f) and (Figure 2a, b, & c). H&E sections from another focus showed carcinoma in situ in the urothelium, in which the thickness of transitional epithelium was normal, the individual cells were malignant, and had lost their polarity through the entire thickness of epithelium. The basement membrane was intact (Figure 2a, b) (Figure 2c). In another focus, the same histological features and possible micro-invasion by carcinoma in situ was seen, with edematous submucosa, chronic inflammation and dilated congested vessels (Figure 2a, b & c), (Figure 3a, b, c, d, & f). Immunohistochemical staining of sections showed intense cytoplasmic positivity for CK 7 (Figure 3, a) and focal positivity for CK 20 indicative of dysplastic change according to P. Hardan et al (12) (Figure 3b). Epstein-Barr virus latent membrane-1 (LMP) stain was negative for EBV (Figure 3c, d) , while immune staining for *P*63 revealed intense and diffuse nuclear staining in the transitional as well as syncytial epithelial cells in LELC (Figure 3e). 

Also, pathological findings and slides from 103 more cases of transitional cell carcinoma were retrieved from pathology archive and reviewed for the above mentioned changes (table 1).

Clinicopathological features of 103 cases of TCC looking for foci of Lymphoepithelial – like carcinoma, grading, muscular invasion, and carcinoma in situ in urothelium (table 1).

**Clinicopathological findings**

Clinicopathological features of cases of Lymphoepithelial–Like Carcinoma of the urinary bladder reported in the most relevant English Medical Literatures were reviewed (table 2)

**Table 1:** Clinicopathological features of 103 cases of TCC reviewed looking for carcinoma in situ, garde, muscular invasion, and focus of LELC

Grade	Age(mean)	CIS	CIS(Pagetoid)	MuscleInvation	Number	LELC
I	55.3	5	-	-	35 33.9	-
II	66.3	10	-	24/1	4344.7%	-
III	70.6	20	5 48%	8	25 24.2%	1
Total	64.2	35	5	10	103	1

**Table 2:** Continued, Clinicopathologic features of the Lymphoepithelial like carcinoma of G. U. Tract reported in world Medical Literatures

Ref. No	No. of Cases	Author’s Name	Gender & Age Range	Pattern of tumor	Recommendation of therapy
	-	Mahul B. Amin	-	Comments on diagnostic , therapeutic and prognostic implications	Pure & Predominant LELC have favorable Prognosis , Focal LELC should be treated with more aggressive procedure
	13	Lopes-Beltran et al	M=9 F=4 67-82	Focal=4 Pure =3 Predominant =6	Favorable prognosis in pure and predominant form cases multi-agents chemotherapy recommended for salvage of bladder function
	1	N.M. Pantelides et al	64 year Male	Focal Dis. More aggressive Diffuse and pure or predominant tumor are less aggressive	Focal radical cystectomy , pure & predominant are less aggressive -bladder preserved , all patients treated with TUR-B & chemotherapy
	1	Antonio B. Procaro et al	72-year old , male	Pure & Predominant has favorable Prognosis , Focal LELC has poor prognosis	Pure & Ptedominent should be treated with TUR-B & Chemotherapy to salvage bladder , patient with focal Dis. Should be treated with radical cystectomy and systemic therapy
	11	Amin MB. et al	52-79	3 pure 8Mixed with LELC and TCC FOCAL (3/8 )	Chemotherapy and TUR-B Potential of salvaging Bladder function
	30	E.F. Tamas et .al	Mean age 67.6 years 21 males (70 %)	Seventeen cases pure 56.7 % 13 cases were mixed , 47 % with TCC	Partial cystectomy would be ill advised
	1	E. R. Kessler et al	65Year- old , female	Pure LELC more responsive to chemotherapy	Pure type of LELC more responsive to chemotherapy

## Discussion

Lymphoepithelial carcinoma is considered to be an undifferentiated carcinoma of the nasopharynx described first at this location, and has bimodal age-incidence (1). Cases have shown to have familial aggregation, genetic predisposition, environmental factor influence and pathogenic link to Epstein –Barr virus (EBV). It has been proposed that this tumor requires EBV genome expression for initiation while the maintenance and progression requires alteration in the cellular genes (1). The treatment of choice for this type of malignancy is a combination of radiation and chemotherapy with 83% 10-year survival (1). Lymphorpithelial–like carcinoma has been described in many organs including the salivary glands, thymus, lung, skin, esophagus, stomach, liver, gall bladder, G.U. tract, uterine cervix and prostate (1),most of which have close pathogenic link with EBV (1). In the salivary glands, lymphoepithelial–like carcinoma has been considered to be a subtype of undifferentiated carcinoma with evidence of familial clustering, which is more frequent among Eskimos and Chinese and has close pathogenic and serologic links with EBV infection, and the overall outcome and prognosis is clearly better than undifferentiated carcinoma(1). In the thymus, lymphoepithelial–like carcinoma has been described and the documentation of EBV genome in some cases suggested that the similarity with nasopharyngeal carcinoma extends beyond the morphological parameters (1). In the lung, lymphoepithelial-like carcinoma is considered to be a subtype of large cell undifferentiated carcinoma and EBV genome has been documented. This tumor is more frequent in Orientals, and the prognosis is better than non–small cell undifferentiated carcinoma of the equivalent stage (1). In the stomach, lymphoepithelial–like carcinoma has been described with strong pathogenic relation to EBV. The EBV expression makes the prognosis better, which is possibly related to the presence of activated cytotoxic T-cell infiltrate (1). In the prostate, it has been described and considered to be a variant of adenocarcinoma (1). In the G.U. tract, Lymphoepithelial–like carcinoma has been seen most commonly in the urinary bladder, but cases have been also reported in renal pelvis, as well as the ureter and urethra. The first report of this carcinoma of the urinary bladder was described by L.R. Zukerberg et al in 1991 (13). In the urinary bladder and renal pelvis, as most authors suggested, Lymphoepithelial–like carcinoma has no pathogenic link to EBV (1). 

The most controversial issue is the approach to and the management of the patient with lymphoepithelial-like carcinoma of urinary bladder. E.F. Tamas et al reported 30 cases of lymphoepithelial–like carcinoma of urinary bladder, renal pelvis and urethra. They divided their cases into pure and mixed cases (with conventional TCC) and found an association of urothelial carcinoma in 47 % and also possibility for multifocality. Accordingly, the partial cystectomy would be typically ill advised for lymphoepithelial– like carcinoma (3) because of chance of multifocality.

On the other hand, some authors believe that the tumor of lymphoepithelial–like carcinoma has a more favorable prognosis compared to conventional urothelial carcinoma, the 5-year actuarial survival for lymphoepithelial–like carcinoma is 59 % (3). Still, others believe that because lymphoepithelial–like carcinoma is more sensitive to systemic chemotherapy and radiotherapy, as has been suggested by Tateki Yoshino et al ( 8 ), TUR-B alone or combined with adjuvant systemic chemotherapy for pure or predominant mixed pattern, as has been suggested by Antonio B. Porcaro et al , and FM Izquierdo – Garcia et al ( 15,&9 ) is recommended. They claim that because the prognosis was very good after radiotherapy and chemotherapy, preserving the bladder despite of the muscle infiltration should be considered (15) . N.M. Pantelides et al suggested that there is a growing evidence for the efficiency of chemotherapy coupled with TUR-B as part of a bladder–preserving treatment option (16). Sten Holmang et al. believed that diffusely pure or predominant lymphoepithelial– like carcinoma had good prognosis and when treated with locoregional therapy there was no evidence of disease for 4 years, while the patient with focal Lymphoepithelial–like carcinoma had a poor prognosis similar to conventional bladder carcinoma of the same size and stage. In these cases, radical cystectomy and systemic chemotherapy are recommended (15). Meanwhile, S. Willamson et al recommended TUR-B with systemic chemotherapy, however; a large–scale study with long term follow–up is recommended (6). In an attempt to try to solve the controversial problem of approach and treatment of the patient with lymphoepithelial–like carcinoma, histologic parameters are the main determinants in the prognosis of this type of malignancy i.e. whether the tumor is pure or and diffuse, has a mixed pattern, focality and/or is associated with carcinoma in situ changes, since carcinoma in situ changes (as seen in six out of 30 cases) has a tendency for multifocality (3). Antonio Lopez – Beltran et al reported 14 % carcinoma in situ in the adjacent urothelium and muscular invasion in 13 cases (5), and Kakizo et al examined cystectomy specimens from 118 cases of Transitional cell carcinoma and they found carcinoma in situ and dysplasia adjacent to and remote from the visible tumor. They found that more than 50% of grade III transitional cell carcinoma were associated with carcinoma in situ and dysplasia in adjacent and remote from visible tumor (11).

 In conclusion, thorough pathologic examination is very important in the prognosis and therapy decision of these patients. By considering the chance of multifocality and also the association with carcinoma in situ, we propose to take multiple biopsies from the mass, adjacent urothelium, and random biopsies from normal looking urothelium to rule out concurrent carcinoma in situ or dysplasia, before proceeding and choosing a definite procedure. This is helpful to see whether the lymphoepithelial–like carcinoma is focal or diffuse, pure or mixed, associated with carcinoma in situ or dysplasia. In case of single pure lymphoepithelial-like carcinoma, prognosis is excellent and therapeutic maneuvers are more bladder saving. In the case of multifocality and association with carcinoma in situ, a more radical approach is recommended. In this regard, immunohistochemical stain for CK 20 is recommended by P. Hardan et al (12), before a definite procedure is being determined, whether it is radical cystectomy, partial cystectomy, or TUR-B and systemic chemotherapy.

## References

[1] Amin MB (2009). Histological variants of urothelial carcinoma: diagnostic therapeutic and prognostic implications. Modern Pathology.

[2] Amin MB, Ro JY, Lee KM, Ordóñez NG, Dinney CP, Gulley ML ( 1994). Lymphoepithelial – Like carcinoma of the urinary bladder. Am J Surg Pathol.

[3] Beltrán AL, Luque RJ, Vicioso L, Anglada F, Requena MJ, Quintero A (2001). Lymphoepithelioma-like carcinoma of the urinary bladder: a clinicopathologic study of 13 cases. Virchows Archiv.

[4] Hardan P, Eardly I, Joyce AD (1996). Cytokeratin 20 as an objective marker of urothelial dysplasia. British Journal of Urology International.

[5] HOLMANG S, BORGHEDE G, Johansson SL (1998). Bladder carcinoma with lymphoepithelioma-like differentiation: a report of 9 cases. The Journal of urology.

[6] Izquierdo-Garcia FM, Garcia-Diez F, Fernandez I, Perez-Rosado A, Saez A, Suarez-Vilela D (2004). Lymphoepithelial – Like carcinoma of the urinary bladder three cases with clinicopathological and P 53 Protein expression study. Virchows Archiv.

[7] Kakizoe T, Matumoto K, Nishio Y, Ohtani M, Kishi K (1985). Significance of carcinoma in situ and dysplasia in association with bladder cancer. The Journal of Urology.

[8] Kessler ER, Amini A, Wilson SS, Breaker K, Raben D, La Rosa FG (2015). Lymphoepithelioma – Like carcinoma of the urinary bladder, Oncology Journal. Bladder Cancer.

[9] Mills SE, Greenson JK, Hornick JL, Longacre TA Reuter VE Sternberg’s Diagnostic Surgical Pathology.

[10] Pantelides NM, Ivaz SL, Falconer A, Hazell A, Winkler M, Hrouda D (2012). Lymphoepithelial – Like carcinoma of the urinary bladder : A case report and review of systemic treatment options. Urology Annals.

[11] Porcaro AB, Gilioli E, Migliorini F, Antoniolli SZ, Iannucci A, Comunale L (2003). Primary lymphoepithelial – Like carcinoma of the urinary bladder report of one case with review and update of literature after a pooled analysis of 43 cases. International Urology and Nephrology.

[12] Rosai J Rosai Ackerman’s surgical pathology.

[13] Stein JP, Lieskovsky G, Cote R, Groshen S, Feng AC, Boyd S (2001). Radical Cystectomy in the Treatment of Invasive Bladder Cancer : Long-Term Result in 1,054 patients. Journal of Clinical Oncology.

[14] Tamas EF, Nielsen ME, Schoenberg MP, Epstein JI (2007). Lymphoepithelioma-like carcinoma of the urinary tract: a clinicopathological study of 30 pure and mixed cases. Modern pathology.

[15] Williamson SR, Zhang S, Lopez-Beltran A, Shah RB, Montironi R, Tan PH (2011). Lymphoepithelioma-like carcinoma of the urinary bladder: clinicopathologic, immunohistochemical, and molecular features. The American journal of surgical pathology.

[16] Yoshino T, Ohara S, Moriyama H (2014). Lymphoepithelioma-like carcinoma of the urinary bladder: a case report and review of the literature. BMC research notes.

[17] Zukerberg LR, Harris NL, Young RH (1991). Carcinomas of the urinary bladder simulating malignant lymphoma A report of five cases. The American journal of surgical pathology.

